# Home Care for the Elderly: An Integrated Approach to Perception, Quality of Life, and Cognition

**DOI:** 10.3390/ijerph21050539

**Published:** 2024-04-25

**Authors:** Luis Eduardo Genaro, José Victor Marconato, Elaine Pereira da Silva Tagliaferro, Felipe Eduardo Pinotti, Aylton Valsecki Júnior, Tânia Adas Saliba, Fernanda Lopez Rosell

**Affiliations:** 1 Postgraduate Program in Collective Health in Dentistry, School of Dentistry, São Paulo State University, Araçatuba 16.015-050, SP, Brazil; felipe.pinotti@unesp.br; 2 School of Medicine, San Francisco University, Bragança Paulista 12.916-900, SP, Brazil; vmarconato@outlook.com; 3 Department of Community Dentistry, School of Dentistry, São Paulo State University, Araraquara 14.801-903, SP, Brazil; elaine.tagliaferro@unesp.br (E.P.d.S.T.); aylton.valsecki-junior@unesp.br (A.V.J.); fernanda.lopez-rosell@unesp.br (F.L.R.); 4 Department of Preventive and Restorative Dentistry, School of Dentistry, São Paulo State University, Araçatuba 16.015-050, SP, Brazil; tania.saliba@unesp.br

**Keywords:** home care services, home nursing, mini-mental state examination

## Abstract

This study investigated the impact of home care, health status, and cognition. A qualitative and quantitative approach was employed through a cross-sectional study with a sample of 60 elderly individuals in need of home care in the municipality of Itatiba, São Paulo, Brazil. The analysis utilized the Discourse of the Collective Subject (DCS), EQ-5D, EQ VAS, and Mini-Mental State Examination (MMSE). The sample consisted of 40.0% male and 60.0% female individuals. The majority (61.6%) received weekly visits, mainly from community health agents, who were responsible for the majority of the care (45%). Positive considerations were highlighted, with 36.6% emphasizing the contribution to treatment continuity. The EQ VAS assessment indicated a moderately good perception of health. The EQ-5D analysis revealed significant differences between genders in personal care (*p* = 0.04). There were significant differences between clinical characteristics and EQ-5D dimensions, such as neoplasia and reduced mobility (*p* = 0.04), and arthritis/osteoarthritis/rheumatism and a limitation in common activities (*p* = 0.01). The presence of anxiety/depression was significant in cases of neoplasia (*p* = 0.006), arthritis/osteoarthritis/rheumatism (*p* = 0.01), and stroke (*p* = 0.04). The logistic regression analysis showed associations between usual activities and arthritis, osteoarthritis, rheumatism (*p* = 0.034), pain/malaise and arthritis, osteoarthritis, rheumatism (*p* = 0.038), and anxiety/depression and stroke (*p*= 0.028). The average MMSE scores (17.52) suggested a mild cognitive impairment, with no statistical differences between genders. Based on these results, it can be concluded that home care can provide a comprehensive approach and continuous assistance, emphasizing the importance of personalized care based on perceived and clinical differences.

## 1. Introduction

Globally, the aging population trend is on the rise. According to the World Health Organization, it is estimated that the proportion of the population aged over 60 will increase from 12% to 22% between 2015 and 2050 [1]. With an aging population, there is a growing demand for supportive care for dependent elderly individuals, catering to the general public [1,2]. The rise in life expectancy and the increase in elderly individuals with chronic illnesses highlight the need for long-term care, becoming a major challenge for healthcare systems in many countries [3].

Recently, an action plan titled “Healthy Aging Decade 2020–2030” was disclosed. This plan calls for concerted, catalytic, and sustained collaboration over a period of ten years [4,5,6]. Its specific objectives include preventing elderly poverty and providing quality education, employment opportunities, and inclusive infrastructure for all age groups. The proposal aims to bring together efforts from governments, civil society, international agencies, professionals, academia, media, and the private sector to enhance the quality of life for the elderly [5,6].

Brazil, the largest country in South America with the fifth-largest global population, faces significant social and economic disparities. With a population exceeding 200 million, aging in the country is happening rapidly. Despite recent improvements in socioeconomic and health indicators, studies show that health inequalities in the elderly have remained unchanged over the last 10 years [7,8].

In the Brazilian context, aging with dignity is recognized as a fundamental human right, supported by the principles of a Democratic Social State. Article 230 of the 1988 Constitution ensures that “family, society, and the state have the duty to support the elderly, guaranteeing their participation in the community, defending their dignity and well-being, and ensuring their right to life” [9]. Currently, three main legal instruments, the Constitution, the National Elderly Policy, and the Elderly Statute, establish and protect the rights of the elderly in the country [8,10].

In 1976, the first Social Policy for the Elderly proposed community participation to help the elderly stay with their families, revising licensing criteria for long-term care institutions, and anticipating the creation of specialized medical services. Additionally, the policy suggested a review of the social security system, retirement preparation programs, professional training for elderly care, and systematic data collection on the elderly population [8,11].

A long-term care policy for the elderly should guide the development of models that consider the ongoing support needed for elderly individuals with varying levels of dependency. This includes coordinated home and community care, services in assisted living and nursing facilities, day care, and palliative care. Recognizing the importance of family care, it is crucial to provide greater instrumental, emotional, and financial support to caregivers. This policy should also regulate caregiving as a profession, coupled with comprehensive training and support, in response to the growing demand for caregivers [12,13].

When it comes to home care for the elderly, current research has focused on meeting needs but has placed less emphasis on the quality of care [14,15,16,17]. The quality of care provided by caregivers is crucial to ensure the well-being of the elderly and the quality of their daily lives. Studies also indicate that the quality of care provided to the elderly is directly related to the professional satisfaction of caregivers [18,19].

In the context of home care for the elderly, there are significant gaps in the provision of assistance services, including medical care, physical therapy programs, spiritual support, cultural activities, and other services beyond basic daily life care [20]. Family members often lack professional nursing knowledge and care tools, and are unable to provide emergency medical treatment for elderly individuals with disabilities [21].

“Good practice” in Brazil was officially established through Board Resolution-RDC No. 63 of 25/11/2011. This resolution recognizes home care for the elderly as a resource that humanizes, controls the transmission of diseases, and contributes to maintaining the health of the elderly in their environment, ensuring care close to their family [22]. Healthcare professionals should act as agents of transformation in society, involving the family in elderly care. An effective approach to promoting health is engaging the elderly in various activities, such as interaction groups and health education [23].

However, uncertainty persists regarding the impact of home care on the health outcomes of the elderly population. While substantial evidence indicates that home care can improve the health of the elderly, promoting a sense of community and maintaining social networks during aging in place [24,25,26,27], concerns exist about the less stringent control of the quality and quantity of home care compared to other forms of care [18,27]. Low-quality home care can result in medical issues and depression in elderly patients [27,28].

Therefore, accurately estimating the impact of home care on the health of the elderly, identifying its effects in different populations, and analyzing the influencing mechanisms are crucial. It is of great importance to assess the current home care system [29,30].

The aim of this study was to investigate the impact reported by the elderly regarding the use of home care. We conducted an analysis of perception regarding home care, health states, and cognition. The results will contribute to expanding the field of home care studies, seeking a more comprehensive understanding of the current impact of this care and providing an essential practical guide to further optimize the provision of this type of service. This, in turn, sheds light on the implementation of future policies related to the growth of home care services.

## 2. Methodology

### 2.1. Study Design and Sample Selection

The sample comprised elderly individuals receiving home care (N = 60) over a period of 1 to 4 years. These participants were selected based on their ability to answer questions during the interview. The study was conducted in the municipality of Itatiba, São Paulo, Brazil. The study was approved by the Human Research Ethics Committee of the Universidade Estadual Paulista (UNESP) of the Faculty of Dentistry of Araraquara (CAAE: 69122923.6.0000.5416). Written informed consent was obtained from participants.

### 2.2. Study Setting

The study took place in the municipality of Itatiba—SP, located in the interior of the State of São Paulo/Brazil, and considered medium-sized. It is part of the Metropolitan Region of Campinas, located northwest of the state capital, approximately eighty kilometers away. Its population as estimated by IBGE in 2019 was around 120,858 inhabitants. The municipality has 12 Family Health Units (USF), which were included in the study.

The life expectancy of Itatiba citizens is 75.6 years, the adult literacy rate is 0.934, and the School Attendance rate is 0.826, with 56% of the population aged 18 to 24 having at least completed secondary education.

In 2021, GDP per capita was BRL 66,360.02. In comparison with other municipalities in the state, they ranked 75th out of 645 among the state’s municipalities and 519th out of 5570 among all municipalities.

### 2.3. Data Collection

Data were collected by a trained researcher, using a self-administered questionnaire and individual semi-structured interviews recorded on a digital recorder. We chose this method because it ensures face-to-face interaction, allowing participants to express their thoughts and arguments spontaneously, in detail, and free from any interference. During the interviews, a script containing questions related to home care was used. To ensure the confidentiality and anonymity of participants, questionnaires were identified with numbering. Data collection took place from October 2023 to January 2024. After the interviews, recordings were transferred from the recorder to the computer for transcription of the speeches for subsequent analysis.

### 2.4. Discourse of the Collective Subject (DCS)

Data analysis was performed through descriptive analysis and analysis based on the qualitative–quantitative technique of Discourse of the Collective Subject (DCS) [31,32], aided by Qualiquantisoft^®^ version 1.3.c, to facilitate the analysis of data from qualitative research. According to Lefevre et al. [32], DCS is based on the theory of Social Representation, its sociological assumptions, and the analysis of verbal material collected from each of the testimonies. It is founded on the belief that within any social group, individuals share ideas, opinions, beliefs, and expressions, and these shared opinions are gathered in a synthesis discourse. This discourse reflects similar contents and arguments among individuals about a specific issue, starting from various individual testimonies and arriving at a collective testimony. Thus, the raw material for this technique comes from the interviews conducted.

The questions were formulated in an open and objective manner, as recommended by Lefevre and Lefevre [31]. After being formulated, the questions underwent pre-testing in a pilot study.

Questions addressed to the participants:

1. How often are home visits conducted in the context of caring for the elderly?
2. Among the professionals of the Family Health Strategy Team, which one performs the highest number of home visits?
3. How do you perceive the home care you receive?

### 2.5. Health-Related Quality of Life Measured with EQ-5D

Scoring on the EQ-5D, which measures Health-Related Quality of Life (HRQoL), involves assessing five main dimensions of an individual’s health status: mobility, self-care, usual activities, pain/discomfort, and anxiety/depression. Each of these dimensions has three levels of response, indicating the presence of problems to varying degrees [33].

Patients are asked to provide responses for each dimension, encoding their condition on a numerical scale. For instance, code 1 may represent “no problems”, code 2 may indicate “some problems”, and code 3 may correspond to “many problems”. The combination of these codes across the five dimensions forms a unique health profile for each individual [33,34].

### 2.6. Value Associated with Health Status

EQ-5D assumes two ways of associating value with a person’s health status. The first, to complete the description of health status, offers the respondent the possibility of locating their own health status on a visual analog scale. Using the direct measurement technique, respondents are asked to draw a line between the ‘box’ representing their health status at that moment and the EQ-VAS thermometer from 0 to 100, considering 0 the worst imaginable health status and 100 the best imaginable health status [33].

### 2.7. Mini-Mental State Examination (MMSE)

The MMSE comprised five test categories: orientation, memory registration, memory recall, calculation and attention, and language. Orientation tests included graded questions about time and place, totaling 10 points. Memory registration asked subjects to remember three unrelated items, and memory recall required them to repeat the items later. Serial calculation was then tested for attention level by subtracting 7 from 100 with five repetitions. Finally, language tasks included naming, repeating, following three-stage commands, reading, writing, and copying designs. The total score ranged from 0 (worst score) to 30 (best score) points [35,36].

### 2.8. Statistical Analysis

The qualitative results were analyzed using the Qualiquantisoft^®^ software version 1.3.c and presented through descriptive analysis and tabulation, considering frequency. Meanwhile, quantitative data underwent a normality analysis through the Shapiro–Wilk test. As the data did not follow a normal distribution, the evaluation was conducted using the chi-square test. Those showing significant differences were subsequently subjected to logistic regression. To summarize quantitative data, measures of central tendency and dispersion were employed, presenting absolute and relative frequencies. In all conducted analyses, a significance level of 0.05 was adopted. The statistical analyses were performed using IBM SPSS Statistics 19.0 (IBM Corp., Armonk, NY, USA).

## 3. Results

Table 1 provides a comprehensive analysis of the sample distribution based on various demographic and health variables. The sample appears to represent a diverse population, allowing for a more in-depth understanding of its characteristics. In this context, some relevant interpretative points stand out.

The sample demonstrated a balanced distribution between genders, with 40.0% male individuals and 60.0% female individuals, suggesting a gender-balanced representation in the studied population.

Regarding age groups, there was a significant concentration between 71 and 85 years (45.0%), with a substantial portion of 35.0% aged 86 and above, highlighting the predominance of older individuals in the sample. In the marital status analysis, the majority were married (43.4%), followed by those in a common-law union (30.0%), while singles represented only 3.33% of the sample. In terms of education, 60.0% had incomplete elementary education, contrasting with 15.0% who completed elementary education and 5.0% with incomplete high school education.

The economic distribution highlighted a predominance in class D/E (90.0%), compared to the lesser representation in class C (10.0%), emphasizing socio-economic differences in the sample. Regarding the duration of home care, the analysis revealed a varied distribution, with 41.6% receiving care for 13–24 months, 21.6% for 1–12 months, and 23.3% for 25–36 months, highlighting the complexity of home care needs.

Finally, the presence of specific clinical characteristics, such as obstructive lung disease (35.0%), heart failure (25.0%), arthritis/osteoarthritis/rheumatism (23.3%), stroke (16.6%), and neoplasia (15.0%), was found.

The data in Table 2 shows that the majority of participants (61.6%) receive weekly home visits. Community health agents are responsible for the majority of care (45%), as reported by the participants, who highlighted the consistent presence of these professionals. Nursing technicians also play a significant role, carrying out visits in 18.3% of cases, focusing on ensuring the general health of participants.

In terms of considerations about home care, a significant group (36.6%) emphasized that the visits contribute to treatment continuity, covering post-surgical cases and wound care. Additionally, a substantial portion (25.0%) highlighted the humanized assistance provided by the professionals. The majority of participants (31.6%) rated the care as good, emphasizing both the frequency and quality of home visits.

Table 3 presents an analysis of the EQ-5D dimensions based on gender, exploring the categories of mobility, personal care, usual activities, pain/discomfort, and anxiety/depression. The data are divided between male and female genders.

In the mobility category, we observed that the proportions of individuals at different levels (1, 2, and 3) varied between genders. Notably, women had a higher representation at levels 2 and 3 compared to men; however, the difference was not statistically significant (*p* = 0.45). 

In personal care, there was a statistically significant difference (*p* = 0.04) between genders. Women showed a more significant representation at levels 2 and 3, indicating a greater need for assistance in this dimension compared to men. A similar result was observed in the usual activities dimension, where women had a more pronounced presence at levels 2 and 3.

Regarding pain/discomfort, there was no significant difference between genders (*p* = 0.12). However, in the anxiety/depression dimension, there was a statistically significant difference (*p* = 0.03). Women had a more significant representation at levels 2 and 3, indicating a higher prevalence of anxiety/depression compared to men.

Table 4 presents an analysis of the EQ-5D dimensions in relation to different clinical characteristics.

The relationship between the clinical characteristics and EQ-5D dimensions revealed a statistically significant difference, such as the presence of neoplasia and reduced mobility (*p* = 0.04) and arthritis/arthrosis/rheumatism associated with limitations in usual activities (*p* = 0.04). 01). The presence of anxiety/depression was statistically significant in cases of neoplasia (*p* = 0.01), arthritis/arthrosis/rheumatism (*p* = 0.006), and stroke (*p* = 0.04).

According to data from Table 5, a statistically significant association was observed between the performance of habitual activities and the presence of arthritis, osteoarthritis, or rheumatism (*p* = 0.034). Similarly, the presence of pain/discomfort also showed a significant association with the occurrence of arthritis, osteoarthritis, or rheumatism (*p* = 0.038). Additionally, a statistically significant association was identified between the presence of anxiety/depression and the occurrence of stroke (*p* = 0.028). These findings underscore the importance of these variables in understanding morbidity associated with specific health conditions, emphasizing the relevance of emotional aspects and daily activities to overall health.

**Figure 1:**
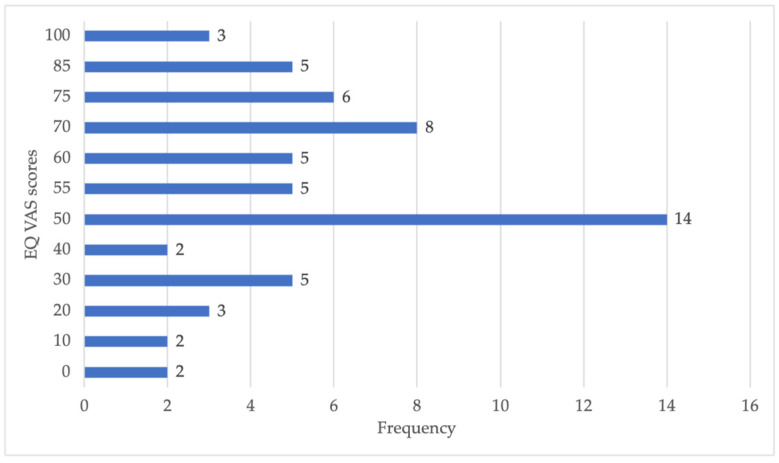
EQ VAS scores midpoint for patients requiring home care.

It is equally enlightening to present the complete distribution of individual data points from the EQ VAS, primarily through a graphical representation. An illustrative example is found in Figure 1.

In this graphical image, not only is the shape and centrality of the distribution trend visualized, but also the range of scoring preferences is understood. A higher frequency of responses is observed at the score of 50, indicating that the self-reported health status by the participants was predominantly considered moderately good.

As seen in Table 6, the range of 18–23 in MMSE is associated with mild cognitive impairment, while scores below 17 suggest significant cognitive impairment. Thus, the average of 17.52 fell within the borderline zone between mildly compromised cognition and significantly compromised cognition. This indicates that, on average, participants may show early signs of cognitive decline, deserving further attention and consideration. There was no statistically significant difference between genders.

## 4. Discussion

Challenges faced by the healthcare system in adapting to the new demographic dynamics, marked by an increase in the elderly population and, consequently, the prevalence of chronic diseases, have raised concerns. Therefore, understanding the specific characteristics of this population will enable the formulation of effective strategies and guidelines for combating and preventing these diseases. The present study aimed to assess the perception of home care, health status, and cognition of the elderly in need of home care.

Regarding the perception of the care provided, the answers indicated different frequencies of visits and professionals who carry out home visits for health care. Some participants mentioned the weekly presence of health professionals, such as nurses and health agents, who check health conditions, advise on medications, and offer support. Others reported less frequent visits, such as every 15 days or monthly, especially after improvements in their health condition. Furthermore, there are a variety of professionals involved, from doctors and nurses to nursing technicians and dentists.

Considerations about home care were predominantly positive, highlighting the importance of visits to maintain treatment, perform dressings, and offer support in specific situations such as those related to mobility difficulties. Some participants expressed gratitude and confidence in the care received, while others suggested that visits could be more frequent to address doubts and ensure a more complete follow-up. In general, experiences reflected the appreciation of home visits as a way to ensure accessible and personalized healthcare.

The results suggest that interventions in the home environment aim to meet fundamental human needs, providing comfort, satisfaction, and restoring the balance of biopsychosocial functions. These needs are vital for life maintenance and are shared by all individuals. However, the approach to satisfying them varies according to the context in which the subjects are inserted, and their implementation is not limited to the individual dimension [37].

In the sample, statistically significant differences were identified in two of the five dimensions of quality of life, as measured using the descriptive component of the EQ-5D. The dimensions related to personal care cover basic and instrumental components of daily activities. The ability to self-manage is a crucial factor for the health and well-being of the elderly, as corroborated by some authors [38].

It is predictable that the loss of functional capabilities impacts the performance of daily activities, generating feelings of additional effort, with implications at personal, social, and economic levels. Previous studies indicate discrepancies between genders, a phenomenon also observed in our research. A possible explanation for the greater disability in women is associated with a higher prevalence of non-fatal disabling conditions, resulting in prolonged survival and making them more prone to the mentioned challenges [39].

The anxiety/depression dimension also revealed significant score differences between men and women, corroborating results from previous studies. These studies indicate that the female gender, somatic diseases, cognitive and functional decline, lack or loss of social contact, and a history of previous depression are key indicators related to depressive disorders and symptoms [39,40].

Evidence indicates disparities in mental health between men and women, highlighting that gender is a psychosocial construct that inevitably influences the expression of mental health [41]; in addition, the mental health condition revealed in the study may be related to the condition of decreased mobility. A proposed explanation is that women are socialized to internalize suffering, contributing to disorders associated with depression, anxiety, and suicidal ideation. In contrast, men are encouraged to act and express their suffering [39,41].

When evaluating clinical characteristics in relation to the EQ-5D, statistically significant differences were observed, especially in the categories of neoplasia, mobility, and anxiety/depression. These factors emerged as significant sources of impaired quality of life in the elderly. Mobility may be correlated with aging [42], while neurobehavioral symptoms, such as depression, often remain underdiagnosed or undertreated in cancer patients [43,44,45]. Additionally, stroke (AVC) was also associated with statistically significant differences in the anxiety/depression category, as evidenced by the logistic regression test (*p* = 0.028). This is due to the substantial occurrence of anxiety in post-stroke patients over time [46,47,48].

There were statistically significant differences among arthritis, osteoarthritis, and rheumatism in various categories, including usual activities (*p* = 0.01), pain/discomfort (*p* = 0.04), and anxiety/depression (*p* = 0.006). This association was confirmed through a logistic regression test, which revealed a relationship between the usual activities condition and arthritis, osteoarthritis, and rheumatism (*p* = 0.034), as well as between pain/malaise and arthritis, osteoarthritis, and rheumatism (*p* = 0.038). These results can be explained due to this ailment being a prevalent degenerative disease that causes pain, joint deformity, and functional disability, and compromises the quality of life [49,50,51].

The EQ VAS scores showed a more significant frequency of responses with a score of 50, indicating that the participants generally perceived their health status as moderately good. To date, it has not been feasible to compare this result with previous studies involving home care patients, and this score may be associated with the specific health conditions of the participants.

Cognitively, there was a slight cognitive impairment observed (mean = 17.52). Mild cognitive impairment is a condition often found in the elderly, characterized by a deterioration in memory, attention, and cognitive function beyond what would be expected based on age and education level, without significantly interfering with the ability to perform daily activities [52,53].

Among the major psychiatric conditions affecting the elderly are depression, bipolar disorder, anxiety, psychotic disorders, and dementia [54,55]. Some of the results presented in this analysis also resembled those obtained in the study conducted by Quadros Junior et al. [55], where the average age of the elderly undergoing cognitive tests was 74.21 years, and the average score on the Mini-Mental State Examination (MMSE) was 18 points.

A limitation of this study can be attributed to the use of a non-probabilistic sample, even though it covered all the elderly individuals in need of home care in the city of Itatiba. This may constrain the generalization of results to the overall population, as the sample may not fully represent the diversity of the elderly population requiring home care. However, given the scarcity of studies on the subject, the current research has the potential to make a significant contribution to the field.

## 5. Conclusions

Based on the data presented, it can be concluded that home care can provide a comprehensive approach and continuous assistance. The prevailing clinical conditions highlight the complexity of the care required for this segment of the population. The weekly frequency of home visits, especially those carried out by community health agents and nursing technicians, highlights the importance of the consistent presence of these professionals in the lives of the elderly, contributing not only to health monitoring, but also to the continuity of treatment, as was emphasized by the participants. Furthermore, the statistical analysis of the DSC, EQ-5D, EQ-VAS, and MMSE instruments provided valuable data on subjective health perception, with significant differences and important associations observed, which reinforced the importance of personalized care.

## Figures and Tables

**Table 1:** Distribution of samples by categories of demographic and health variables.

	Variables	n	%
Gender			
	Male	24	40.0
	Female	36	60.0
Age Group (years)			
	65–70 years	12	20.0
	71–85 years	27	45.0
	>86 years	21	35.0
Race/color			
	White	18	30.0
	Black	22	36.6
	Brown	20	33.4
Marital Status			
	Single	2	3.33
	Married	26	43.4
	Divorced	6	10.0
	Widowed	8	13.3
	Common-law union	18	30.0
Education			
	No education	5	8.3
	Incomplete elementary school	36	60.0
	Complete elementary school	9	15.0
	Incomplete high school	3	5.0
Economic class			
	C (monthly household income of R$ 2.9 thousand to R$ 7.1 thousand)	6	10.0
	D/E (monthly household income up to R$ 2.9 thousand)	54	90.0
Home care duration			
	1–12 months	13	21.6
	13–24 months	25	41.6
	25–36 months	14	23.3
	37–48 months	8	13.3
Clinical characteristics			
	Obstructive Lung Disease	21	35.0
	Heart Failure	15	25.0
	Neoplasia	9	15.0
	Stroke	10	16.6
	Arthritis/Osteoarthritis/Rheumatism	14	23.3

Some participants exhibit one or more clinical characteristics.

**Table 2:** Summary of Discourse of the Collective Subject (DCS) questionnaire results organized by categories associated with each question.

	n	%	Discourse of the Collective Subject
Frequency of Home Care Visits			
Weekly	37	61.6	“Usually, it’s every week, they come and check, ask if I’m taking the medicine”, “Every week, they show up at home, check my blood pressure, and guide me on how to take care of myself”.
Once every 15 days	20	3.3	“Look, they show up at least once every 15 days”, “Every 15 days, they come here at home to ask if everything is fine”.
Once a month	3	5.0	“Now that I’m no longer hospitalized, they come once a month to my home. When I was really unwell, they used to come more often, but now everything is fine”. “The doctor comes here from time to time, I think it’s once a month”.
Which professional conducts more home visits			
Community Health Agent	27	45.0	“The person who comes most often to our home is the health agent; she helps explain how to take the medicine”. “Every week, the agents come here to inquire if we need anything from the health center, even asking if we have enough food”.
Nursing Technician	11	18.3	“A young lady and a young man always come to my house. If I’m not mistaken, they are nurse technicians”. “Here at home, it’s usually the technicians; it’s harder for the doctor to come, only when I really need it”.
Nurse	9	15.0	“The people from the health center who come here are usually a nurse”. “Normally, a nurse comes to visit here at home to make sure everything is in order with my health”.
Doctor	9	15.0	“The doctor comes to the house regularly to check if everything is okay”. “It’s the doctor who comes more often to the house to fix the prescription for the medicine”.
Dentist	2	3.3	“Now, the dentist is coming more to clean the teeth. They were quite dirty, so he comes to clean”. “The last few times, it was the dentist from the health center who came home”.
Physiotherapist	2	3.3	“The physiotherapist is coming here to help with walking”. “It’s more the doctor who helps with getting up that comes to the house”.
Considerations about Home Care			
Treatment continuity	22	36.6	“Oh, their visits help us to maintain the treatment, you know. I had surgery last week, and the girls come to dress the wound”. “It’s good to continue the consultation... I can’t go to the health center because, look, I’m bedridden, can’t walk”.
Humanized assistance	15	25.0	“The people from the health center are like angels; I had difficulty walking, and they already arranged for a doctor to help me get up”. “They know what they’re doing, and they always ask if they can help. You can see their affection for us, you know”.
Good care	19	31.6	“I think it’s good; they come here every week”. “Look, I have nothing to complain about. In the other city where I lived, nobody came to the house. Here, they come and provide very good care”.
Could be more frequent	4	6.6	“I like that they come to my house, but they could come more often. They only appear from time to time”. “They could come more often; the doctor comes occasionally, and it’s very quick. Then, I’m left with a lot of doubts”.

**Table 3:** Analysis of EQ-5D dimensions by gender.

EQ-5D Dimension	Gender				
	Male		Female		*p*
	N	% (IC95%)	N	% (IC95%)	
Mobility					
1	4	11.1	6	25.0	
2	16	44.4	8	33.3	
3	15	41.6	11	45.5	0.45
Personal Care					
1	8	22.2	3	12.5	
2	12	33.3	14	58.3	
3	7	19.4	16	66.6	0.04 *
Usual Activities					
1	8	22.2	4	16.6	
2	13	36.1	16	66.6	
3	9	25.0	10	41.6	0.42
Pain/Discomfort					
1	2	5.5	3	12.5	
2	21	58.3	12	50.0	
3	8	22.2	14	58.3	0.12
Anxiety/Depression					
1	15	41.6	6	25.0	
2	11	30.5	18	75.0	
3	4	11.1	6	25.0	0.03 *

* The Chi-square test revealed a statistically significant difference (α = 0.05).

**Table 4:** Relationship between clinical characteristics and EQ-5D dimensions.

EQ-5D Dimensions	Clinical Characteristics									
	Obstructive Lung Disease		Heart Failure		Neoplasia		Stroke		Arthritis/Osteoarthritis/Rheumatism	
Mobility		*p*		*p*		*p*		*p*		*p*
1	4		5		-		-		1	
2	12		4		2		6		4	
3	4	0.06	6	0.10	7	0.04 *	4	0.22	9	0.13
Personal Care										
1	5		5		-		-		2	
2	12		6		3		6		3	
3	3	0.06	4	0.17	6	0.13	4	0.20	9	0.09
Usual Activities										
1	6		5		-		-		1	
2	12		3		5		7		4	
3	3	0.14	7	0.12	4	0.24	3	0.16	9	0.01 *
Pain/Discomfort										
1	2		2		-		-		1	
2	14		7		4		6		4	
3	4	0.16	6	0.63	5	0.34	4	0.44	9	0.04 *
Anxiety/Depression										
1	9		8		-		1		1	
2	9		4		5		5		12	
3	2	0.13	3	0.09	4	0.01 *	4	0.04 *	1	0.006 *

* The Chi-square test revealed a statistically significant difference (α = 0.05).

**Table 5:** Logistic regression analysis of variables with statistical significance in EQ-5D in relation to morbidity.

Variables		Model	
	β	Z	*p*
Mobility × Neoplasia	0.265	0.505	0.613
Usual activities × Arthritis, osteoarthritis, rheumatism	1.087	1.238	0.034 *
Pain/malaise × Arthritis, osteoarthritis, rheumatism	1.157	0.973	0.038 *
Anxiety/depression × Neoplasia	1.0746	1.715	0.086
Anxiety/depression and stroke	0.9057	1.134	0.028 *
Anxiety/depression × Arthritis, osteoarthritis, rheumatism	−0.979	−1.916	0.055

* Statistically significant difference, α = 0.05.

**Table 6:** Mean performance and variation in MMSE scores and subitems by gender.

	Scores		Male	Female	
	Mean	Standard Deviation	Mean	Mean	*p*
MMSE (total)	17.52	2.77	17.52	17.50	0.75
Subitems					
Temporal orientation	3.54	0.97	3.52	3.51	0.67
Spatial orientation	3.32	1.14	3.27	3.25	0.58
Immediate memory	2.34	0.70	2.32	2.32	0.88
Attention and calculation	2.10	1.30	2.00	2.01	0.97
Evolutionary memory	1.12	0.74	1.13	1.08	0.38
Naming	1.44	0.49	1.40	1.43	0.93
Repetition	0.60	0.49	0.59	0.56	0.13
Command	1.82	0.70	1.80	1.79	0.39
Reading	0.54	0.50	0.47	0.50	0.45
Sentence	0.52	0.50	0.50	0.51	0.53
Drawing	0.45	0.52	0.47	0.48	0.75

## Data Availability

The raw data supporting the conclusions of this article will be made available by the authors on request.
